# The ontology of fast food facts: conceptualization of nutritional fast food data for consumers and semantic web applications

**DOI:** 10.1186/s12911-021-01636-1

**Published:** 2021-11-09

**Authors:** Muhammad Amith, Chidinma Onye, Tracey Ledoux, Grace Xiong, Cui Tao

**Affiliations:** 1 School of Biomedical Informatics, The University of Texas Health Science Center at Houston, 7000 Fannin St, Suite 600, Houston, TX 77030 USA; 2 Surveillance and Epidemiology Unit, Office of Surveillance, Science, and Technology, Harris County Public Health, 2223 West Loop South, Houston, TX 77027 USA; 3 Department of Health and Human Performance, University of Houston, 3875 Holman St. Rm 104 Garrison, Houston, TX 77204 USA; 4 Department of Neuroscience, University of Texas, 110 Inner Campus Drive, Austin, TX 78705 USA

**Keywords:** Ontology, Semantic web, Fast food, Nutrition, Micropublishing

## Abstract

**Background:**

Fast food with its abundance and availability to consumers may have health consequences due to the high calorie intake which is a major contributor to life threatening diseases. Providing nutritional information has some impact on consumer decisions to self regulate and promote healthier diets, and thus, government regulations have mandated the publishing of nutritional content to assist consumers, including for fast food. However, fast food nutritional information is fragmented, and we realize a benefit to collate nutritional data to synthesize knowledge for individuals.

**Methods:**

We developed the ontology of fast food facts as an opportunity to standardize knowledge of fast food and link nutritional data that could be analyzed and aggregated for the information needs of consumers and experts. The ontology is based on metadata from 21 fast food establishment nutritional resources and authored in OWL2 using Protégé.

**Results:**

Three evaluators reviewed the logical structure of the ontology through natural language translation of the axioms. While there is majority agreement (76.1% pairwise agreement) of the veracity of the ontology, we identified 103 out of the 430 statements that were erroneous. We revised the ontology and publicably published the initial release of the ontology. The ontology has 413 classes, 21 object properties, 13 data properties, and 494 logical axioms.

**Conclusion:**

With the initial release of the ontology of fast food facts we discuss some future visions with the continued evolution of this knowledge base, and the challenges we plan to address, like the management and publication of voluminous amount of semantically linked fast food nutritional data.

## Background

Fast food or “limited service” restaurants provide foods that are mass-produced and served quickly. These establishments allow for food and drink to be consumed on-premises, taken out, or delivered to the customer, and offer a wide selection of food choices. Healthier menu offerings include salads and lean grilled meats yet pizza, hamburgers, and fries remain the most commonly purchased items. Over the past 3 decades, Americans have increased their intake of foods prepared outside the home and currently, 36% of US adults eat at fast-food restaurants each day [1]. These rates are similar in children with one-third of US children consuming fast food each day [2].

Fast foods are popular among people of most age groups due to their low cost, consistency, and convenience [3]. The majority of research shows that fast food consumption is linked to excess weight gain, poor diet quality, and mortality. Recent research suggests that improvements in nutrition labeling have resulted in the availability of healthier items for consumers to choose from [4].

The following literature review examines the effect of fast food on individual health and the impact of nutrition labeling on consumer health outcomes.

### Impact of fast food on health

Food is essential for every human being and the quality of the food consumed has a significant impact on an individual’s health. Diet quality is based on adherence to national nutritional recommendations and dietary guidelines that promote health, meet nutrient needs, and prevent disease. Certain types of fast foods can lead to excess weight gain, where a change in weight status occurs that causes an individual to be categorized as overweight or obese according to body mass index. Excess weight gain puts people at risk for developing diseases and conditions that increase one’s likelihood of death or mortality due to cardiovascular diseases. Some research suggests that living in areas that are densely populated with fast food restaurants can impact individual health due to the increased accessibility of fast food for consumption, and also position the motivation for the development of a consumer-centric ontology of fast food nutritional information.

#### Excess weight gain

The overall results of the studies indicate that there is an association between the consumption of fast-food and excess weight gain. A prospective cohort study conducted by [2] among 541 pre-school-age children found that weight status increased in children who consumed fast food more frequently during the week.

Of the studies conducted among adults Bhutani and colleagues [5] reported a significant positive relationship between the frequency of eating at fast-food restaurants and increased BMI among 1,418 individuals in a cross-sectional study.

#### Fast food density

A systematic review examined 31 articles to look at the relationship between retail food establishments around schools and the occurrence of overweight and obesity in school aged children [6]. Fourteen studies observed a direct association between proximity or density and excess weight.

Similarly, a systematic review conducted by William et al. [7] examined 20 articles to observe the associations between the retail food environment and body weight. 72 associations were observed with 43 showing a positive relationship. Nineteen of the positive relationships were significant. The authors concluded that there was some evidence of the retail food environment having an effect on children’s bodyweight.

Mazidi et al. [8] conducted a cross-sectional study that evaluated the association between the neighborhood density of fast-food restaurants and obesity prevalence among neighborhood residents. The authors initially observed a negative association which they attributed to the confounding variables of affluence and education. Once the co-founding variables were removed there was no association found between the density of fast-food restaurants and obesity prevalence.

An ecological analysis conducted aimed to examine the relationship between fast food density and the prevalence of type 2 diabetes among counties in South Carolina [9]. The author’s found a significant negative association between fast-food restaurant density and prevalence of type 2 diabetes. The authors found these results to be unexpected and cited individual behavioral decisions as affecting the prevalence of type 2 diabetes.

Another cross-sectional study explored whether an association existed between mortality from stroke or cardiovascular disease and fast-food density in the United States [10]. The authors found that increased fast-food density was associated with an increased risk of death from both stroke and cardiovascular disease along with an increased prevalence of type 2 diabetes. While it was concluded that an association existed it was also determined that the impact of opening a new fast-food restaurant was exceedingly small.

Despite the large number of studies, the findings are mixed and there is limited evidence to suggest that a relationship exists between the food environment and individual health.

#### Diet quality

The evaluation conducted by Barnes and associates examined the association between fast-food consumption and diet quality among working adults [11]. The authors’ determined that there was a significant inverse association between the frequency of fast-food consumption and diet quality.

A cross-sectional study conducted by Vercammen and colleagues examined 1479 combination meals offered by 34 US fast-food and fast-casual restaurant chains [1]. Three options were examined for each combination meal (1) default (2) low-calorie option and (3) high-calorie option. The meals were found to be high in sodium, calories, sugar, and saturated fat. The authors concluded that nearly all the combinations exceeded the daily recommended limits for calories and sodium.

Todd et al. [4] found that in 2013–2014 working adults had greatly increased the amount of fast food they were consuming and yet were experiencing a decrease in their intake of saturated fat and cholesterol by significant amounts. The authors believe these decreases in saturated fat and cholesterol despite higher intake of fast food may suggest an improvement in the quality of fast foods. The authors attributed the improvements to regulations regarding menu labeling beginning in 2008. This may indicate that menu labeling can lead to improvements in the quality of fast food. The authors offer that menu labeling improves consumers’ ability to recognize low-energy food items and suggest that this may compel restaurants to reformulate their menu items by lowering their energy content [4].

#### Mortality

In a prospective cohort study conducted among 69,582 adults increased fast-food consumption was associated with mortality [12]. It was also observed that there was an association between increased fast-food consumption and cardiovascular disease-specific mortality.

### Impact of nutritional information on health outcomes

Access to nutritional information impacts the way people manage their health through diet. Individuals with chronic diseases can monitor their intake of nutrients such as sodium and sugar to slow the progression of disease, while those without chronic illnesses can use nutritional information for disease prevention. Nutritional information also influences decisions regarding what foods people choose to buy and eat.

#### Food management

A prospective cohort study conducted by Amuta-Jiminez et al. [13] observed a relationship between healthy dietary behaviors and the use of food labels among adults diagnosed with cancer. The findings in the study suggest that individuals that used food labels were more likely to engage in health eating behaviors such as consuming more fruits and vegetables and consuming fewer sodas. The findings were significant for all 3 dietary behaviors. A cross-sectional study by Byrd et al. [14] found that consumers that reported taking action to reduce their sodium intake are more likely to use menu nutrition information compared to consumers that reported taking no action to decrease sodium intake.

A randomized controlled trial performed by Kollannoor-Samuel et al. [15] found that the use of food labels leads to improved diet quality and improvements in blood glucose control among adults with type 2 diabetes. The findings were statistically significant at the between-individual level.

A cross-sectional study among 1817 adults conducted by Christoph et al. [16] found that the use of nutritional information was associated with both healthy and unhealthy behaviors involving weight control. The authors also found that these nutrition facts were positively associated with binge eating in women and negatively associated with intuitive eating in men. Christoph et al. [16] suggest that men who use external cues such as nutritional information when choosing food may be less likely to pay attention to internal cues while consuming food. Contrastingly, women may rely on both or neither of these cues when choosing or consuming food. The authors touch on earlier evidence indicating that individuals with eating disorders and weight concerns may be notably influenced by exposure to menu labeling.

#### Food choices

Byrd et al. [17] observed that adding calorie information along with the numeric sodium content of meals to menus resulted in both beneficial and detrimental outcomes. The authors found that consumers who perceived lower calorie and lower sodium foods as tasty were more likely to choose a meal lower in sodium than those who perceive higher calorie and sodium foods as tasty.

Kollannoor-Samuel et al. [18] conducted a prospective cohort study with 12,686 youths and young adults. The authors hypothesized that the use of nutritional labels would be associated with a decreased risk of diabetes in young adults who were not reported to have diabetes at baseline. The authors found evidence that suggested the use of nutrition labels was associated with a lower long-term risk of diabetes.

A randomized controlled trial conducted by Musicus et al. [19] found that red stop sign and traffic light warning labels were associated with a significant decrease in the amount of sodium ordered. The authors also found that the use of warning labels increased knowledge about sodium content and increased abilities to distinguish between high and low sodium meals even without labels.

*Review summary* Due to convenience and availability, more Americans are relying on fast food as a part of their regular diet. Despite the fact that nearly all fast-food restaurants have health promoting items on their menus (e.g., salad), the most commonly purchased items contain excessive amounts of saturated fat, sugar, and sodium. Frequent consumption of fast food has been related to excess weight gain, poor diet quality, and increased mortality risks. However, some research suggests tools such as nutritional labels may empower consumers to make healthier fast-food choices.

While fast food restaurants have incorporated healthier menu items in recent years, studies have shown that there is a link between fast food consumption and poor diet quality. This is attributed to the high levels of sodium, sugar, calories, and saturated fat within menu items. In addition, this poor diet quality associated with fast food consumption is linked to excess weight gain as well as increased mortality risks related to cardiovascular disease.

### Ontologies and big data

Noted earlier nutritional labels, or more generally nutritional information and facts, that can be presented to the health consumers is one factor that can be integral to their decisions to make healthy food decisions. Given the amount of fast food establishments, in addition to non-fast food restaurants, health consumers have a variety of options to potentially decide on nutritional and diet choices. This is also compounded with the amount of choices that fast food restaurants have, emerging new fast food restaurant chains and independent venues (*e.g*., “food trucks”).

We focus this work on how to handle the heterogeneous and volume of fast food nutritional data, and methods to collate the vast amount of evolving data to be available and query-able. Fundamentally, this is a Big Data topic that shares some features of Big Data (*velocity*, *volume*, and *variety*). We expect due to the market demand that fast food items (along with the nutritional information) will change and also increase with more choices from the individual restaurants and from emerging establishments (*velocity* and *volume* features of Big Data). In addition, We assume that with limited amount of nutritional information presented, if the nutritional data was linked to other external extended data sources, the amount of data would predictably increase further. For many restaurants, the nutritional information is presented in varying formats—static and dynamic websites, PDF downloads, siloed websites, etc., and there also regional menu options to accommodate a segment of the world population, but with no accessible solution to aggregate the information for analysis and decision making (*variety* in Big Data).

In this paper we propose the use of an ontology that can facilitate linked data of nutritional information, and provide methods to query the data across the heterogeneous fast food nutritional sources. Ontologies are software artifacts referred to as “formal, explicit specialization of a shared conceptualization” [20]. In a recent article, Hitzler elaborates that “an ontology is really a knowledge base (in the sense of symbolic artificial intelligence) of concepts (that is, types of classes, such as ’mammals’ and ’live birth’) and their relationships (such as, ’mammals give live birth’), specified in a knowledge representation language based on formal logic” [21].

Simply, ontologies utilize symbolic terminologies (e.g. “mammals” and “live birth”) to represent concepts (unit of thought) and first order predicate logic (e.g. “give”) to imbue consistent meaning between concepts. Abstractly this generates a network graph of domain information from the relational links between the concepts. Semantic technology like OWL2 and RDF support authoring of the ontologies and a machine-based syntax for machines to share and interpret standardized knowledge of a domain. Within the framework of the Big Data features, ontologies address data *variety* through standardizing and normalizing heterogeneous data sources and linking to other sources, *velocity* with flexibility to change schema to accommodate fast growth of data, and also *volume* through semantic web technologies like nanopublications.

We discuss the development of an ontology for fast food data and information that aims to normalize and standardize the knowledge of fast food nutritional information. We label this ontology, the ontology of fast food facts (OFFF). OFFF is based on the structure of open-sourced consumer-centric nutritional information presented on fast food websites. Aside from formalizing and having a shared conceptualization of fast food nutritional knowledge, the availability of this knowledge base contributes to future use cases that can potentially benefit health consumers and expert-class researchers and clinicians.

### Related food ontologies

While our research focus is solely aimed for the fast food domain, there are a few “general” food ontologies. The most prominent is FoodOn [22], a BFO (basic formal ontology)-based ontology that has an expansive knowledge base covering various aspects of food knowledge, including the agricultural origin of individual food items. Another is a basic application ontology (Food Ontology) from the British Broadcasting Corporation’s Ontologies for food and recipes [23]. The Recipe Ontology [24] is an application ontology for a serious gaming project. This ontology models rules and information related to recipes and supports personalized player profiles. The Food Ontology Knowledge Base [25] is a basic model of food nutritional information for basic food types from the Republic of Turkey’s Ministry of Food database. The Food and Agriculture Organization of the United Nations provides a linked terminology for expert researchers that covers over 35,000 concepts called AGROVOC [26]. The Food Product Ontology [27] extends the well-known GoodRelations ontology [28] to represent concepts for food products, its pricing, and the associated business entity. Its purpose is to help companies publish and share their food product items using a formal, standard model. Open Food Facts [29] is a crowd-sourced and free terminology source of international food product that rely on volunteers, yet, it is presumed that it may be prone to potential errors due to its crowd-sourced approach and lacks any mechanism to verify the information [30]. Thailand-based Food-Oriented Ontology-Driven System for food menu items is an application ontology for a nutritional recommender system for individuals with diabetes. Similarly for recommender systems, FoodKG [31] is an big data knowledge graph that incorporates a large recipe dataset, the United States Department of Agriculture’s National Nutrient Database for Standard Reference, and the FoodOn ontology. While not a food ontology, the Ontology of Nutritional Studies [32] is another BFO-based ontology that intends to normalize terminologies of nutritional studies to advance data analysis purposes for expert researchers.

### Research objective

The ontology of fast food facts is focused on the pertinent information that health consumers are concerned about, reflected in nutritional labels of fast food. In addition, we represented the knowledge gathered from basic questions that health consumers inquire to enrich the ontology further. In the later sections, we discuss how our work with this ontology will be used as a linked accessible data source, and for patient-facing tools that leverage our previous ontology-based technology for dialogue systems (“chat bots”). Lastly, more specifically, this ontology is solely focused on modeling fast food data and their corresponding nutritional information. This paper discusses the enrichment of our initial effort [33] with expanding and improving the model and further enriching the ontology to address basic information needs of the health consumer that our initial effort did not incorporate.

## Methods

**Fig. 1:**
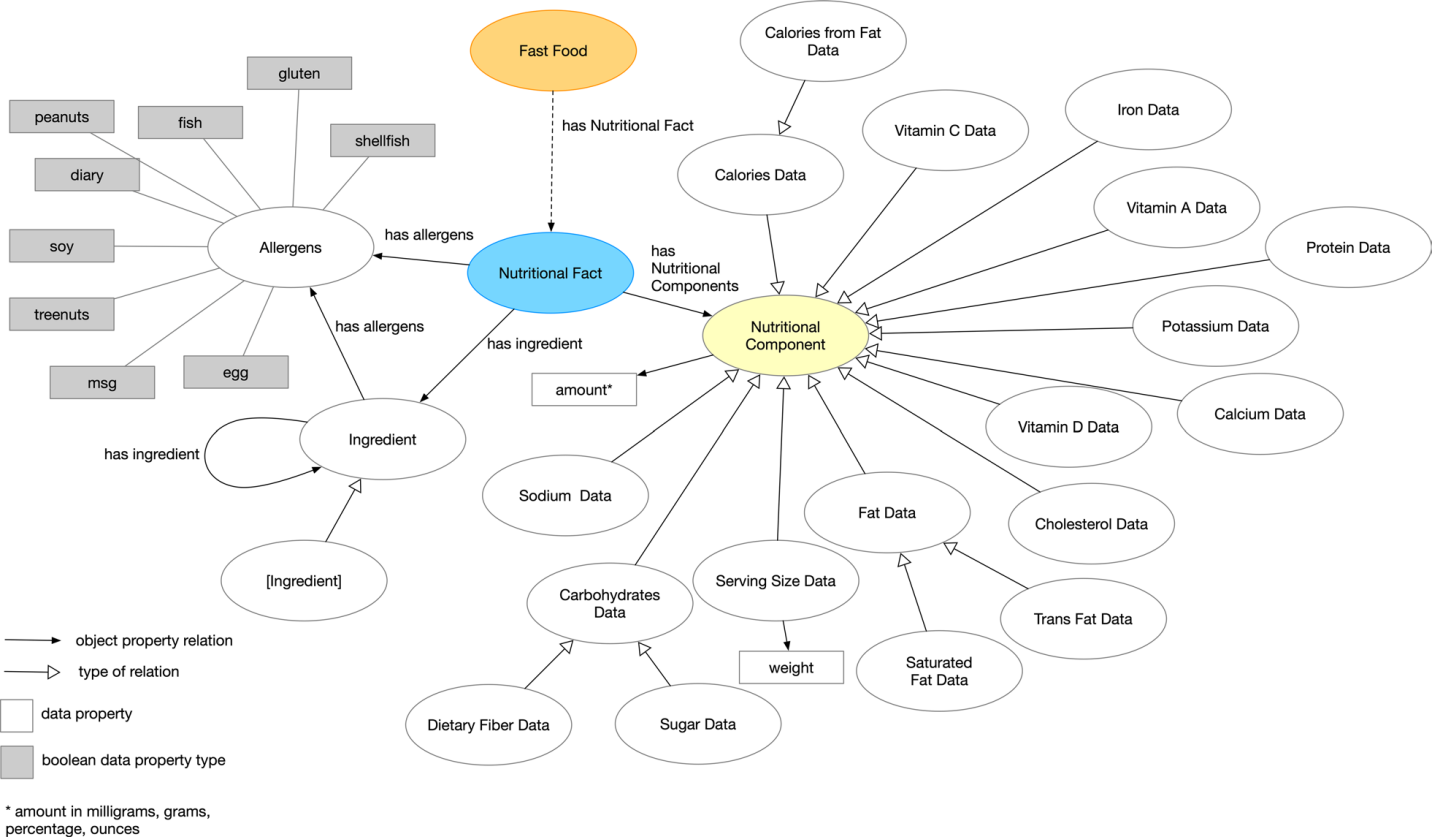
Meta-level of the ontology of fast food facts showing the representation of the *Nutritional Fact* concept and its relationship with the *Fast Food* concept. Dark yellow node indicates additional subclasses

To initiate the design of our ontology, we analyzed consumer-centric nutritional content from fast food establishments—(McDonalds, Dairy Queen, Chick Fil-A, Wendy’s, Taco Bell, Arby’s, Blimpie, Carl’s Jr, Checkers & Rally’s, Church’s, Jack in the Box, Jollibee, Popeye’s, Raising Cane’s, White Castle, and Panda Express). Their websites provide nutritional data either as a web page or downloadable PDF. We devised the meta-data structure from the sources and identified main concepts and relationships between the concepts using the Food and Drug Administration’s standard national nutritional label as a guide [34]. This activity yielded the meta-level abstraction of the ontology (See Fig. 1).

**Fig. 2:**
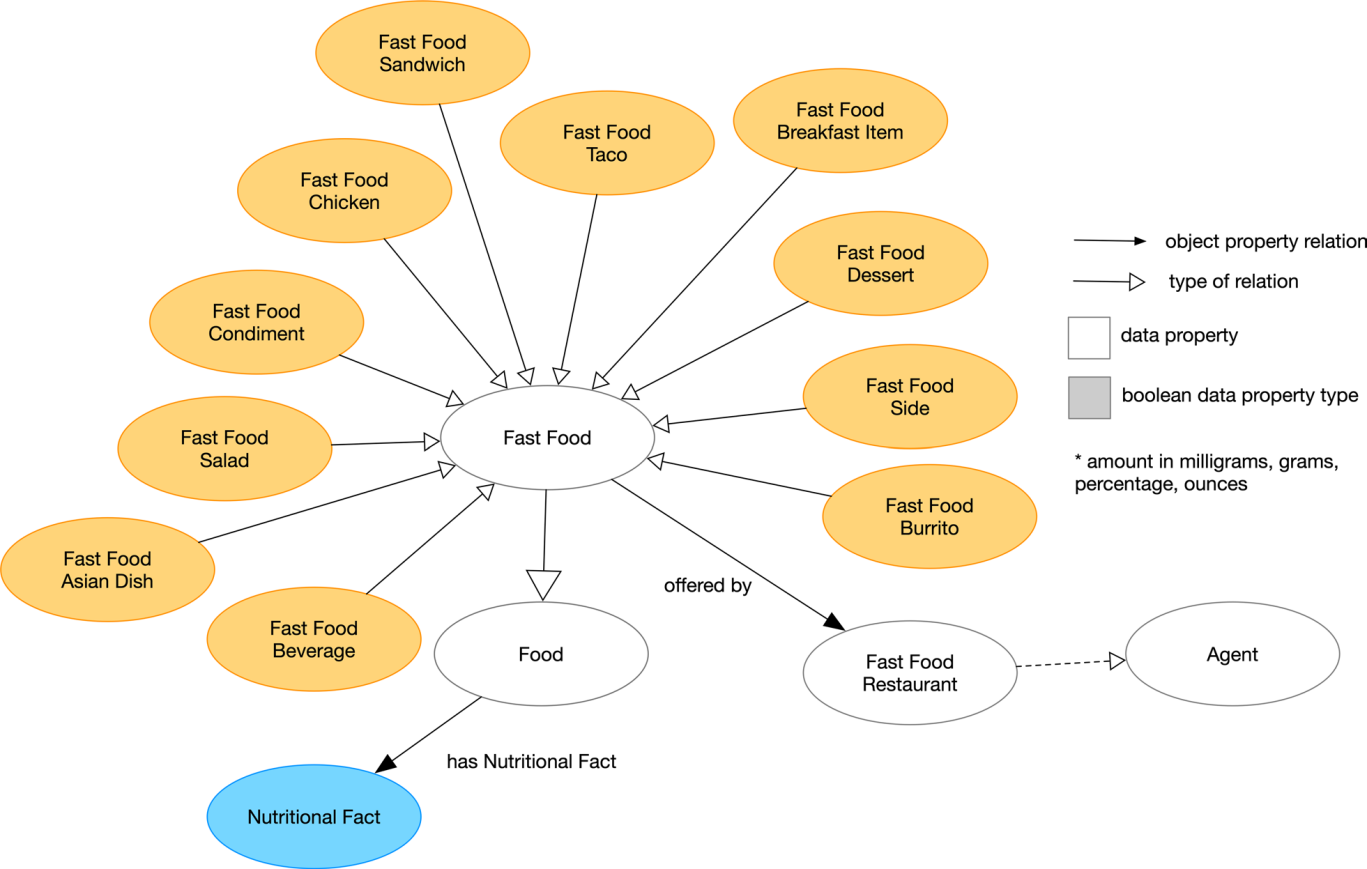
Detailed Meta-level of the Ontology of Fast Food Facts showing the sub-categories for *Fast Food*. Dark yellow node indicates additional subclasses

The central concept of the meta-level of this ontology is *Nutritional Fact*. This concept contains relationships with *Nutritional Component*, *Ingredient*, and *Allergens*. The meta-level of the ontology was authored using Protégé [35]. Later, we evaluated the veracity of the meta-level structure of the ontology using Hootation [36], a natural language generation software library for ontologies. Three evaluators (CO, GX, and CT) independently reviewed the translated natural language statements ($Shredded\_Chicken\_Burrito \subseteq Burrito$ translated to “*every shredded chicken burrito is a burrito*”) that expresses each logical axiom encoded in the ontology. Each evaluator was given a spreadsheet with the translated axioms, and were instructed to label the statements—“Yes” whether the logical axiom is accurate, “No” if the axiom is not accurate, or “X” if the evaluator was unsure.

### Nutritional component

The *Nutritional Component* has several subclasses alluding to various nutritional data found on food labels—protein, cholesterol, fats, minerals, etc. Essentially,*Nutritional Component* describes the amount of nutrients that a particular food item has. This is expressed through a data property of *Amount* (in milligram, gram, ounces, etc.) We also denote amounts of vitamins and minerals contained in a food item—iron, vitamin A, calcium, potassium, etc. This was limited to the vitamins and minerals that are disclosed from the online sources.

### Allergens

*Nutritional Fact* includes allergen information that is represented as *Nutritional Fact* $\rightarrow$ *has allergens* $\rightarrow$ *Allergens*. In many examples from the online sources, allergen content is denoted as a binary representation (i.e., yes or no if a food item has gluten, diary, etc.). The *Allergens* concept has several Boolean data types to flag the food item for allergen content (soy, msg, egg, diary, etc).

### Ingredient

Ingredient information for food items is represented in the ontology with *Nutritional Fact* $\rightarrow$ *has ingredients* $\rightarrow$ *Ingredient*. The *Ingredient* class covers any component listed from the nutritional data such as bread, eggs, corn syrup, caffeine, etc. This concept also has a self-directed relationship *has ingredients* if certain ingredients contained other ingredients. For example, bread can contain milk and eggs (*e.g.*, *bread* $\rightarrow$ *has ingredients* $\rightarrow$ *milk* and *bread* $\rightarrow$ *has ingredients* $\rightarrow$ *egg*). Lastly, the *Ingredient* concept has a link to *Allergens* using *has allergens*. If a food item has milk, it can be indicated with having an allergic reaction of diary.

### Fast food concept

The meta-level, described above, serves as a framework for consumer nutritional information. For fast food items, we created a class called *Fast Food* that subclass an entity of *Food*. This concept is used to describe the types of fast food in most of the fast food establishments. We also included a concept called *Fast Food Restaurant* (linked to *Fast Food* through *offered by*) which is a descendant class of *Agent*, following how other ontologies like PROV-O (Provenance ontology) [37] and FOAF (Friend of a friend ontology) [38] represent organizations ( *Agent* > *Organization* >*Business* ). Based on our limited selection of fast food venues, we identified 10 basic fast food categories (See Fig. 2)—*Fast Food Sandwich *, *Fast Food Taco*, *Fast Food Chicken*, *Fast Food Condiments*, *Fast Food Salad*, *Fast Food Beverage*, *Fast Food Burrito*, *Fast Food Sides*, *Fast Food Breakfast*, *Fast Food Desserts*, and *Fast Food Asian Dish*. *Fast Food* is linked to *Nutritional Fact* with the object property of *has Nutritional Fact* to associate fast food items with nutritional data.

### Instance data model

The nutritional data items from the menus of the fast food establishments were represented as unique instances for the ontology. The aforementioned fast food establishments has nutritional facts either on the website or as a PDF download. We transferred the data from the sources to a spreadsheet for import. We used the Protégé plugin, Cellfie [39], to preform the mass import of the food data. We created a set of custom import rules for Cellfie to normalize and add the data to the ontology.

**Fig. 3:**
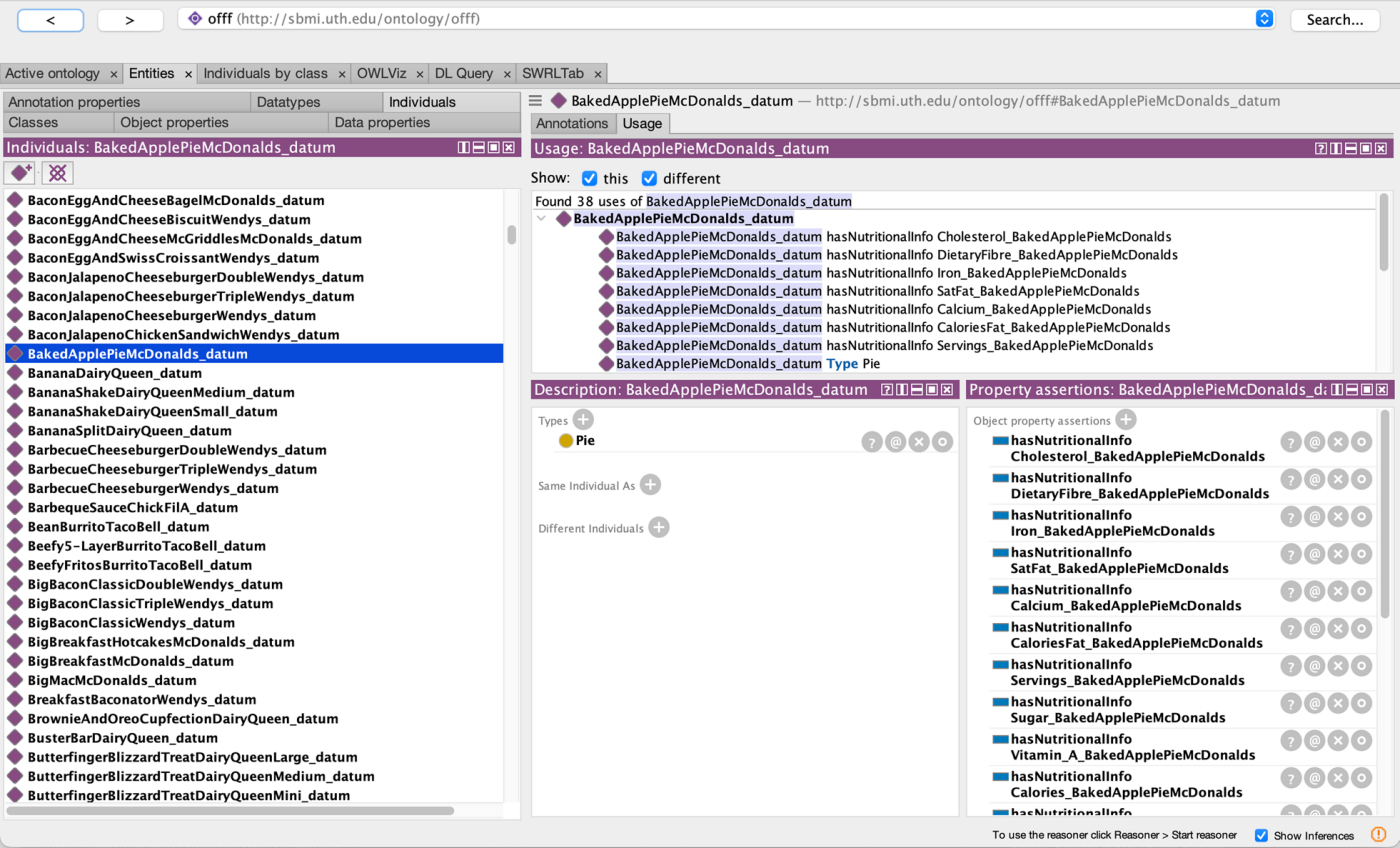
Instance level of the Ontology of Fast Food Facts. Screenshot shows an example of an imported instance for an apple pie item from a major fast food company

**Fig. 4:**
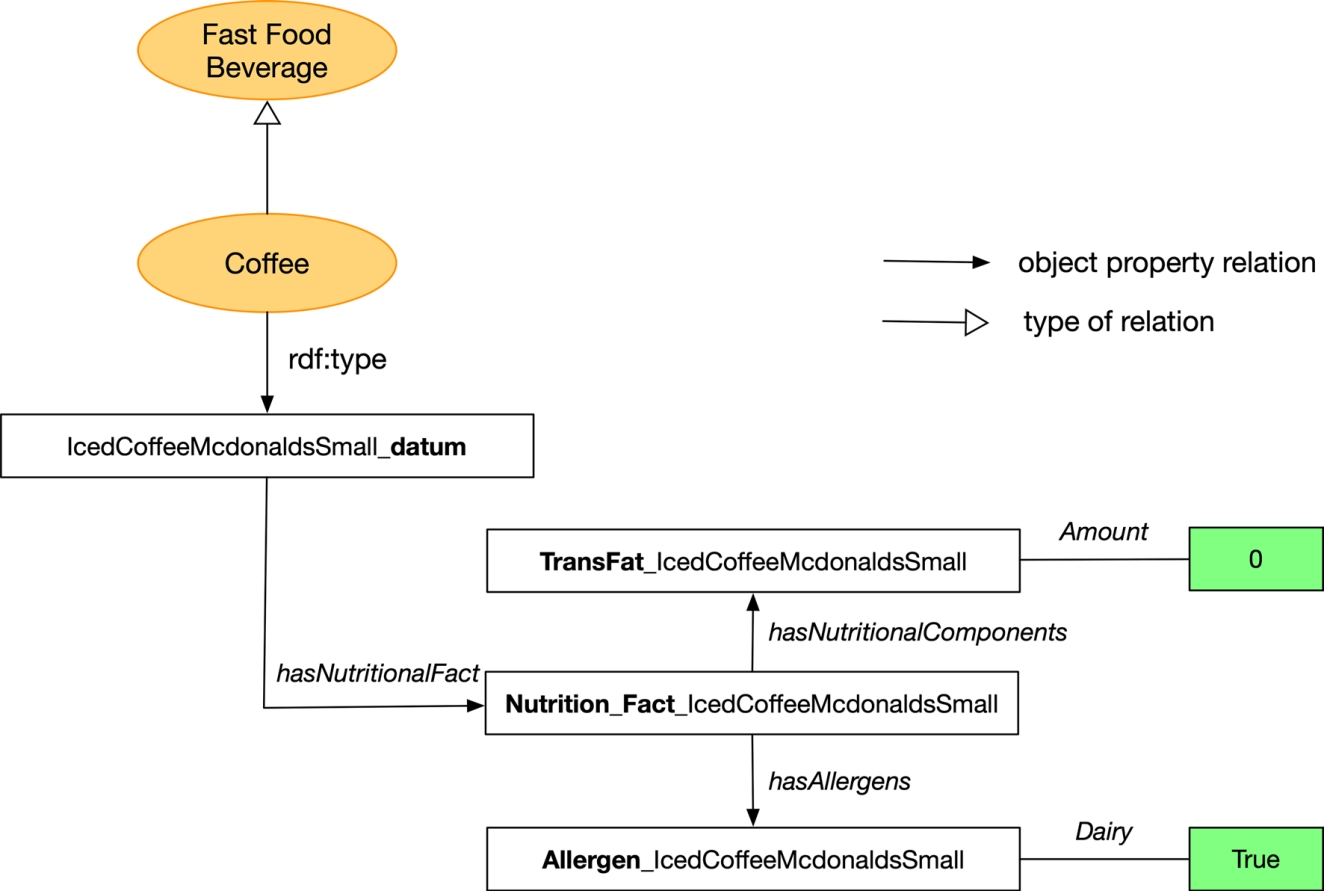
Visualization of a sample instance from the ontology of fast food facts. Suffix and prefix are bold-faced to highlight the type of instance information

Figure 3 shows an example of a final imported data from the collected data, and Fig. 4 visualizes a sample instance data from the Ontology of Fast Food Facts. The food item instantiates the type of fast food. In the example above, Iced Coffee McDonald’s (Small) is an instance of *Coffee* which is a type of *Beverage*. This data item links to other nutritional and allergen information using *hasNutritionalInfo* and *hasAllergens*, as shown in Fig. 4. In the example, trans fat information (*TransFat_IcedCoffeeMcDonaldsSmall*) is associated with the aforementioned iced coffee, and it also indicates the amount of trans fat. The same is with allergen information (*Allergen_IcedCoffeeMcDonaldsSmall*) denoting there is diary allergen. This instance for iced coffee has *_datum* appended to it, and for the nutritional information there is a corresponding prefix (*Allergen_*, *TransFat_*, etc.).

### Enrichment From common nutrition questions

Consumer nutrition questions were gathered by performing Google web searches using the phrases “Frequently asked nutrition questions” and “Most common nutrition questions”. Six sources were selected, each of which listed frequently asked nutrition related questions that were answered by registered nutritionists or registered dieticians. The sources included Consumer Reports [40], The Washington Post [41], North Dakota State University [42–44], The U.S. Department of Agriculture [45], The European Food Information Council [46], and Harvard School of Public Health [47]. The eight sources resulted in 41 questions that were narrowed down to include 19 questions that were used to expand the ontology. Table 1 lists the final 19 questions which were related to outcomes associated with sugar, sodium, and fats.

**Table 1:** 19 Consumer questions used to expand the ontology of fast food facts

Consumer nutrition questions	**Source**
Is fruit bad for me because it contains sugar?	Harvard School of Public Health
Is sugar (or salt or fat) the biggest problem in our diets?	Washington Post
Are naturally occurring sugars healthier than added sugars?	European Food Information Council
Can sugars cause overweight and obesity?	European Food Information Council
Does sugar cause diabetes?	European Food Information Council
Can sugars damage your teeth?	European Food Information Council
How much sugar is OK in a day?	USDA
How many servings from each food group do I need each day?	USDA
How much of a nutrient is too much?	USDA
What are the current recommendations related to fats in the diet?	North Dakota State University
Why should I be concerned about my trans fat intake?	North Dakota State University
Why is “good cholesterol” (HDL) good and “bad cholesterol” (LDL) bad?	North Dakota State University
What are some common sources of trans fat and saturated fat?	North Dakota State University
What is sodium?	North Dakota State University
How much sodium is in table salt?	North Dakota State University
Why should I be concerned about my sodium intake?	North Dakota State University
How much sodium should I have each day?	North Dakota State University
How do I reduce sodium in my diet?	North Dakota State University
What are the common sources of sodium?	North Dakota State University

**Fig. 5:**
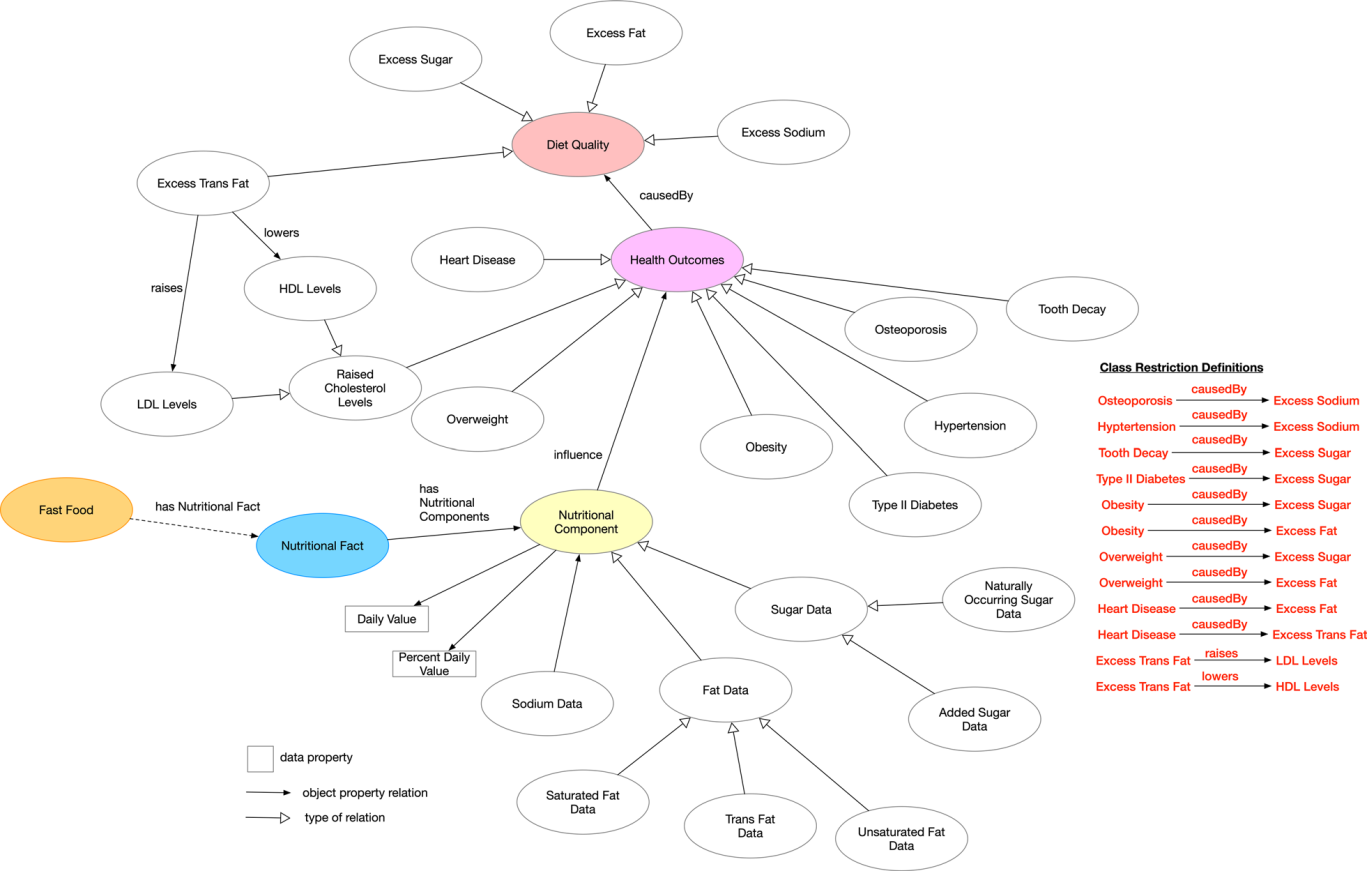
A concept map outlining the additional concepts derived from the consumer nutrition questions. For readability purposes, the class restriction definitions are listed

Figure 5 models the additional concepts and relationships to OFFF. This included the addition of two new concepts: *Health Outcomes*, and *Diet Quality* which were extended through the existing concept of *Nutritional Component*. This also included additional subclasses of the aforementioned concepts, all of which are shown in Fig. 5. These derivations were encoded into the ontology of fast food facts using Protégé.

#### Health outcomes

The *Health Outcomes* concept consists of subclasses that represent various adverse health outcomes that are associated with diet quality. These outcomes include obesity, tooth decay, hypertension, type 2 diabetes, osteoporosis, overweight, heart disease, and raised cholesterol levels. *Health Outcomes* is linked to *Nutritional Component* using *Influence*.

#### Diet quality

The *Diet Quality* concept consists of subclasses that represent attributes identified as contributing to a poor quality diet—excess trans-fat, excess fat, excess sugar, and excess sodium. Each subclass is linked to a subclass of the *Health Outcomes* concept using the object property *causedBy*, *raises* and *lowers*. Figure 5 lists the class restrictions for these subclasses.

#### Daily value and daily value percentage

The *Daily Value* data property expresses the daily recommended value of a nutritional component. *Nutritional Component* is extended using the *Daily Value* data property (positive integer value). Similarly, we also included the percentage of the *Daily Value* (*Daily Value Percentage*) as a decimal value type.

## Results

The ontology of fast food facts (OFFF) contained 413 classes, 21 object properties, 13 data properties, and 494 logical axioms. The three evaluators independently annotated 430 natural language statements. We annotated each statement in terms of (0) whether the statement was not accurately expressed by the ontology and (1) whether the statement was accurately expressed by the ontology. Statements that elicited a response of “don’t know” were annotated as (0). The evaluators achieved substantial intercoder reliability with an average pairwise percent agreement of 76.1%. Pairwise agreement was 73% for raters 1 and 2, 84.7% for raters 2 and 3, and 70.7% for raters 1 and 3. Intercoder reliability was calculated using ReCal3 0.1 Alpha for 3+ Coders.

In total average among the raters, our evaluators assessed that the ontology accounted to 73.0% accurate number of statements (mean of 56.5%, 81.2%, and 81.2%). To our knowledge, we assume that most of the conceptual level structure is accurate. However, we reviewed the statements that had complete disagreement (all evaluators believed or were not sure the statement was accurate) or majority disagreement (two of the three evaluators believed or were not sure the statement was accurate). We accounted 103 statements in all that fit this criteria, and we reviewed the issues with these statements to assess the source of the inaccuracy of OFFF.

We noted three types of issues with OFFF’s accuracy—(1) poor labeling of the entities that could have benefited with more elaborate labels for better expression of the axiom, (2) mislabeling that expressed information that did not reflect the world (e.g., $Mocha \subseteq Coffee\_Fact$, “*every mocha is a coffee fact*”), and 3) logical errors and possible confusion and contention where the issue was not the label but an issue with association of the statement that may have led to confusion or misunderstanding (e.g., $DQ\_Treatzza\_Pizza \subseteq Cake$, “*every dqtreatzza pizza is a cake*”). For the third case, there were 24 statements that fit that category. For the first and second case, there were 48 and 31 statements respectively.

We revised the ontology based on these majority disagreements of the veracity. From our preliminary work, the model of our ontology represented polyhierarchical of fast food facts instead of representing them as entities of food. We oriented the ontology model to reflect a sound organization of fast food, and this is reflected in the design we discussed earlier. This addressed most of the second issue of mislabeling. With issues revolving around more elaboration of the labels, we added more expressive labels by adding terms, like modifying $Taco \subseteq Salad$ (“*every taco is a salad*”) to $Taco\_Salad \subseteq Salad$ ($\approx$ “*every taco salad is a salad*”). For the third issue there were a combination of authoring errors, like duplicate yet erroneous statements, and misunderstanding of the accuracy of the statement. For authoring errors, we had a statement like $Mushroom\_Swiss\_Burger \subseteq Mushroom$ (“*every mushroom swiss burger is a mushroom*”), which were later deleted due to being a duplicate and an error. There were statements that relied on particular knowledge of the food item from a certain restaurants (like Dairy Queen and Whataburger) that used their own nomenclature (e.g., $Apple\_Bites \subseteq Apple\_Slices$ or $Hash\_Brown\_Sticks\_Whataburgers$). Also for the third issue, there were statements that might have been accurate depending on prior understanding the definition of the concepts. For example, OFFF has $Hot\_Dog$ as a type of $Fast\_Food\_Sandwich$—if one were to understand that a sandwich is a breaded food item with some type of non-bread filling. Another example is *Pretzel* or $Oatmeal\_Bar$ as type of $Fast\_Food\_Side$ may be true if there was prior knowledge that these items were available as side item with the entree. All in all, we removed any specific concepts that were vendor-specific nomenclature to ensure normalization and accuracy of the OFFF. The latest version of this ontology is available at the git repository link, https://github.com/UTHealth-Ontology/OFFF.

## Discussion

We developed an ontology called the ontology of fast food facts (OFFF) that models nutritional data of fast food items. This initial work was seeded from nutritional sources published from McDonald’s, Dairy Queen, Chick Fil-A, Wendy’s, Taco Bell, McDonalds, Dairy Queen, Chick Fil-A, Wendy’s, Taco Bell, Arby’s, Blimpie, Carl’s Jr, Checkers & Rally’s, Church’s, Jack in the Box, Jollibee, Popeye’s, Raising Cane’s, White Castle, and Panda Express. With these sources we were able to model the meta-level abstraction that expresses a model of nutritional data and fast food, and then later import the source data onto the conceptual meta-level structure of the ontology with reusable Cellfie scripts. We evaluated the accuracy of the nutritional knowledge in the ontology, and determined if the ontology fulfills the requirements of the competency questions. Most of the knowledge of the nutritional data were accurate, but only about three-fourths of the competency questions were answered with “Yes”.

While the ontology is derived from numerous open-sourced resources from fast food establishments, the ontology is still relatively broad and may need further elaboration of concepts. For example, $Soft\_Drinks$ and *Hamburger* concepts will need additional specific sub-concepts and perhaps class-level restriction definitions to describe “creative” fast food items. Another example is $Breakfast\_Platter$ which is likely to include portions of other fast food items, which signals that this class and others like it will need to be expanded to be more descriptive. There is also unconventional fast food venues, like “food trucks” and emerging multi-ethnic fusion venues, that may have unique or ethnic offerings that could challenge the model of the Ontology of Fast Food Facts. We also do not account for regional offerings (e.g., McDonald’s in Japan) that have items that could expand the model further. We foresee this ontology to further evolve with the next few iterations towards a standard ontology model of fast food that can link nutritional data for analysis and decision making.

**Fig. 6:**
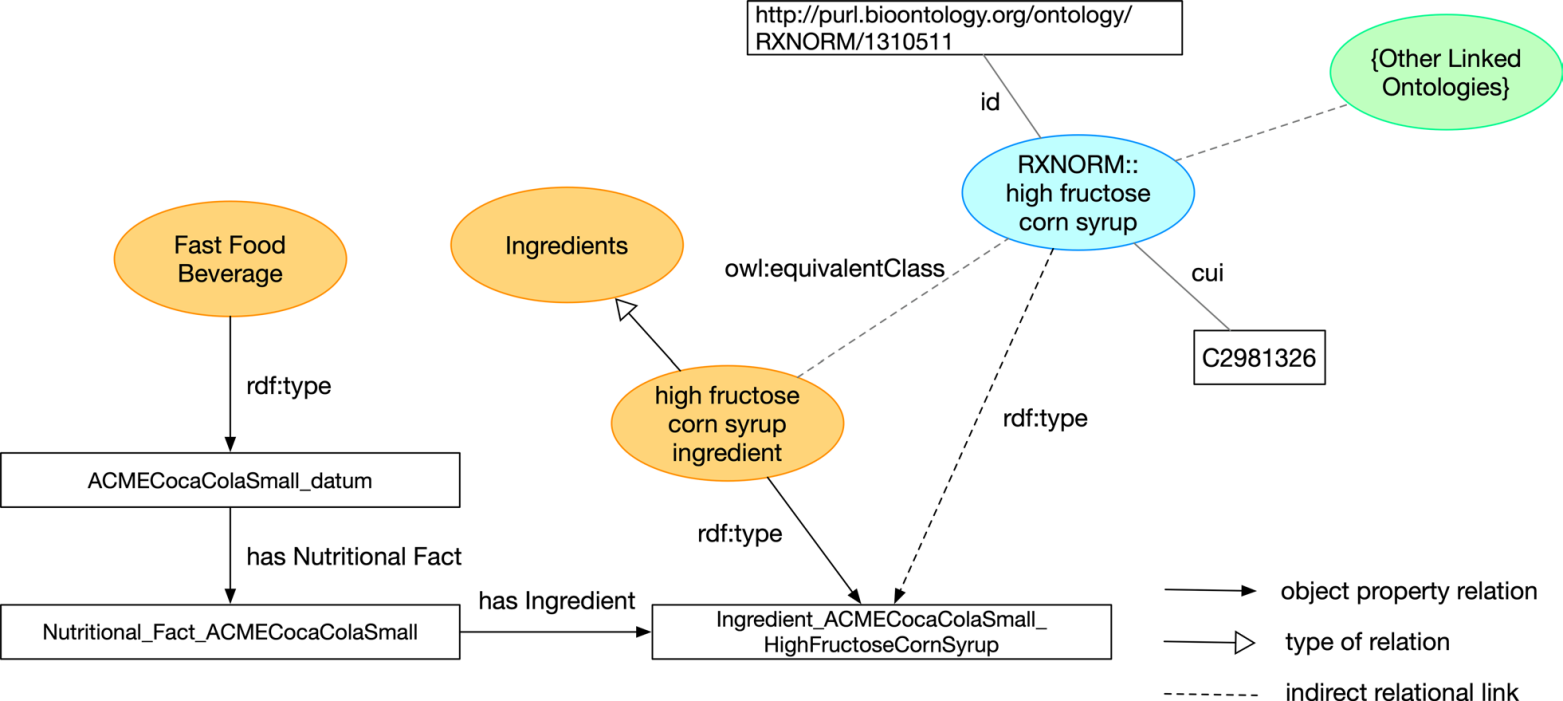
An example displaying concepts from the Ontology of Fast Food Facts linking to other ontologies like RxNORM

A noted feature of ontologies is interoperability, to link and extend data sources. There exist certain concepts that enable OFFF to be link to other ontologies. For example, in Fig. 6 we have one of the Ingredients concept, “high fructose corn syrup ingredient” is linked as an equivalent class to the RxNORM’s [48, 49] version of “high fructose corn syrup” to show how an ingredient of a fast food data item (like a soft drink) can be extended with existing knowledge bases of other ontologies. Furthermore assuming RxNORM is extended and linked to other ontologies it can further elaborate more meaning to a fast food ingredient leading to possible analytical and research possibilities for clinical informatics studies. In addition there is an opportunity in linking to biomedical ontologies with the chemical nutritional entities of OFFF that could enhance future analytical endeavors. A major food ontology, the FoodOn ontology [22], aims to be a comprehensive, realist model of food information ranging from processing of food, distribution, packaging, cooking processes, physical attributes, etc. FoodOn is an ontology for expert level research on food and agriculture. We foresee the possibility of utilizing OFFF to link to FoodOn for traceability analysis for fast food information and also possibly extend the scope of OFFF.

**Fig. 7:**
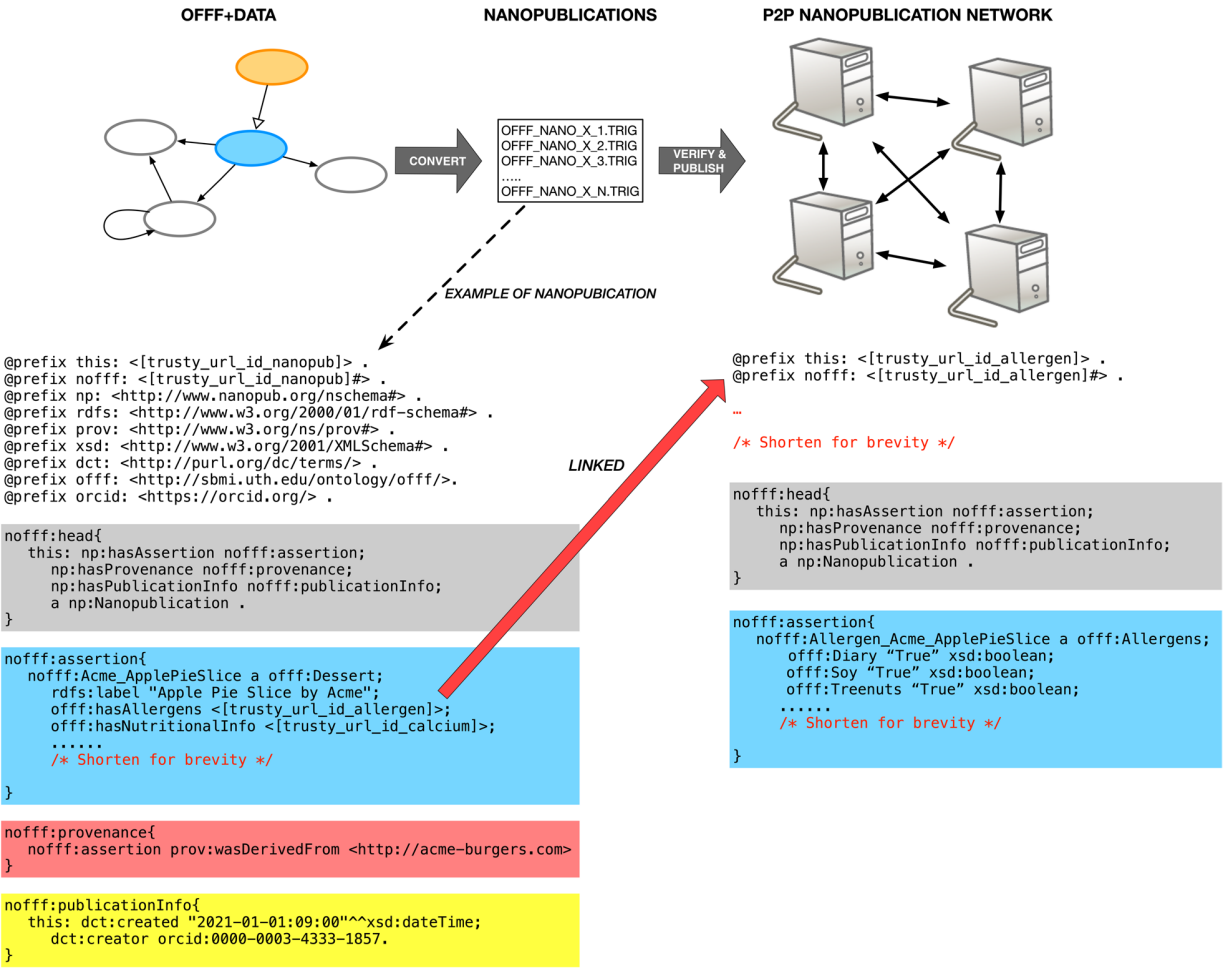
A summary of future proposed publishing pipeline to facilitate the conversion of facts from the Ontology of Fast Food Facts to nanopublication files for distribution

Our challenge we have encountered is the management of imported data. Currently, we have collected nutritional data from 21 sources. The import process toward a merged ontology with the data was predicted to be large and unwieldy based on imports of six of the 21 dataset sources. Our upcoming goal with this work is to push most of the data for public accessibility and consumption. One potential possibility we plan to investigate is to publish nutritional data as nanopublication named graphs and have the data as a persistent store on peer-to-peer network.

The vision of the Semantic Web entails data linked across servers of the World Wide Web to provide meaningful description of data [50]. Portions of the web, like the Open Linked Data Cloud, are early realizations of the Semantic Web vision. Nanopublications builds upon on the vision of the Semantic Web but with a focus on linked scientific assertions over a peer-to-peer decentralized network. A nanopublication is “defined as a small data container consisting of three parts: an assertion part containing the main content in the form of an atomic piece of formally represented data (e.g., an observed effect of a drug on a disease); a provenance part that describes how this piece of data came about (e.g., how it was measured); and a publication info part that give meta-information about the nanopublication as a whole” [51].

With our modeled nutritional data on the nanopublication servers there is a an opportunity to have a reliable, persistent, and queryable source to utilize the fast food nutritional information [51, 52]. Our immediate goal is to develop a custom publication pipeline that will convert our ontology of fast food facts into atomic nanopublication graph formats (.trig) that are linked together. Figure 7 summarizes this next step where a populated OFFF with instance data is decomposed to nanopublication graph formats. Each nanopublication is composed of four basic parts—a header content (gray part of Fig. 7), the assertion content that have one fast food fact (blue-ish part of Fig. 7), the provenance content (reddish-part of Fig. 7), and the nanopublication metadata (yellow part of Fig. 7). Each nanopublication will be assigned a Trusty URI [53], a cryptographic hash value that are immutable and permanent identifiers of digital content to identify the nanopublication of a fast food fact from OFFF. This identifier will also be utilized to link associated data. In the example of Fig. 7, the nanopublication is linked to the allergen information, and potentially to other nutritional data and metadata. We intend with the proposed pipeline to have formalized publication procedure where when there is new data, it will automatically be converted to verified before public release. Subsequently with published fast food nanopublications, we will investigate tools for querying and management which is lacking but of interest with nanopublication research and development [54].

In a separate domain, we (MA, CT) have worked on interactive patient-facing technology for vaccine education and counseling. One of the tools from that endeavor is a dialogue system engine [55] that we envision to automate lightweight evidence-based nutritional counseling to assist in curbing eating behaviors in individuals who have diseases caused by diet and nutritional factors. We intend to use the Ontology of Fast Food Facts to supplement nutritional information that could furnish fast food nutritional information in the automated interactive dialogue. Supplementing the dialogue system engine was a question-answering (QA) subsystem that responded to health consumer questions and generated simple natural language responses from the vaccine knowledge base’s triples [55]. While we recognize that merely answering questions will not impact behavior the way formalized evidenced-based counseling would, we also plan on repurposing the technology to assess the portability and performance of our QA system.

## Conclusion

Poor eating habits either from overeating or food choices has a possible impact on the onset of serious life threatening disease and impacting the quality of life of patients. There is research that alludes that the presentation of nutritional information could have an impact on healthy eating behavior. Yet the copious amount of nutritional information along with potential extended data, complicates any effort to aggregate and centralize nutritional information for consumers and experts to utilize.

We embarked on the development of an ontology of fast food data that aims to normalize and standardize heterogeneous data sources of fast food information, and facilitate high volume and rapid changing amount of nutritional fast food data. The ontology was reviewed to assess the logical construction of the polyhierarchical conceptual level of fast food and fast food nutritional data. While majority of our reviewers perceived most of the ontology’s knowledge to be accurate, a small minority of statements suffered from improper labeling that skewed the logic of the embedded axioms. We revised the ontology and it is available at https://github.com/UTHealth-Ontology/OFFF. This ontology serves as a first step toward potential future direction to completely realize a persistent queryable, public source of linked consumer-centric nutrition data of fast food.

## Data Availability

The ontology of fast food facts generated during the current study are available in the Github repository, https://github.com/UTHealth-Ontology/OFFF.

## Abbreviations

OFFF: Ontology of fast food facts
PROV-O: Provenance ontology
FOAF: Friend of a friend
BFO: Basic formal ontology
QA: Question-answering
